# Early management of patients with aneurysmal subarachnoid hemorrhage in a hospital without neurosurgical/neuroendovascular facilities: a consensus and clinical recommendations of the Italian Society of Anesthesia and Intensive Care (SIAARTI)

**DOI:** 10.1186/s44158-021-00012-9

**Published:** 2021-10-26

**Authors:** Edoardo Picetti, Maurizio Berardino, Alessandro Bertuccio, Rita Bertuetti, Edoardo Pietro Boccardi, Anselmo Caricato, Carlo Alberto Castioni, Marco Cenzato, Arturo Chieregato, Giuseppe Citerio, Paolo Gritti, Luca Longhi, Costanza Martino, Marina Munari, Sandra Rossi, Nino Stocchetti, Tommaso Zoerle, Frank Rasulo, Chiara Robba

**Affiliations:** 1 Department of Anesthesia and Intensive Care, Azienda Ospedaliero-Universitaria di Parma, Parma, Italy; 2 Anesthesia and Intensive Care Unit, AOU Città della Salute e della Scienza, Presidio CTO, Turin, Italy; 3 Department of Neurosurgery, “SS Antonio e Biagio e Cesare Arrigo” Hospital, Alessandria, Italy; 4 Department of Anesthesia, Intensive Care and Emergency Medicine, Spedali Civili University Hospital, Brescia, Italy; 5 Department of Interventional Neuroradiology, ASST Grande Ospedale Metropolitano Niguarda, Milan, Italy; 6 Department of Anesthesia and Critical Care, IRCCS A. Gemelli University Polyclinic Foundation, Rome, Italy; 7 Department of Anesthesia and Intensive Care, IRCCS Institute of Neurological Sciences of Bologna, Bologna, Italy; 8 Department of Neurosurgery, ASST Grande Ospedale Metropolitano Niguarda, Milan, Italy; 9 Neurointensive Care Unit, Department of Neuroscience and Department of Anesthesiology, ASST Grande Ospedale Metropolitano Niguarda, Milan, Italy; 10 School of Medicine and Surgery, University Milano–Bicocca, Milan, Italy; 11 Department of Anesthesia and Critical Care Medicine, Papa Giovanni XXIII Hospital, Bergamo, Italy; 12 Anesthesia and Intensive Care Unit, AUSL Romagna, Bufalini Hospital, Cesena, Italy; 13 Anesthesia and Intensive Care, Padua University Hospital, Padua, Italy; 14 Neuroscience Intensive Care Unit, Department of Anesthesia and Critical Care, Fondazione IRCCS Ca’ Granda Ospedale Maggiore Policlinico, Milan, Italy; 15 Department of Pathophysiology and Transplantation, University of Milan, Milan, Italy; 16 Anesthesia and Intensive Care, San Martino Policlinico Hospital, IRCCS for Oncology and Neurosciences, Genoa, Italy; 17 Department of Surgical Sciences and Integrated Diagnostics, University of Genoa, Genoa, Italy

**Keywords:** Subarachnoid hemorrhage, Management, Transfer, Spoke center

## Abstract

**Background:**

The immediate management of subarachnoid hemorrhage (SAH) patients in hospitals without neurosurgical/neurointerventional facilities and their transfer to a specialized center is challenging and not well covered in existing guidelines. To address these issues, we created a consensus of experts endorsed by the Italian Society of Anesthesia and Intensive Care (SIAARTI) to provide clinical guidance.

**Methods:**

A multidisciplinary consensus panel composed by 19 physicians selected for their established clinical and scientific expertise in the acute management of SAH patients with different specializations (anesthesia/intensive care, neurosurgery and interventional neuroradiology) was created. A modified Delphi approach was adopted.

**Results:**

A total of 14 statements have been discussed. Consensus was reached on 11 *strong recommendations* and 2 *weak recommendations*. In one case, where consensus could not be agreed upon, no recommendation could be provided.

**Conclusions:**

Management of SAH in a non-specialized setting and early transfer are difficult and may have a critical impact on outcome. Clinical advice, based on multidisciplinary consensus, might be helpful. Our recommendations cover most, but not all, topics of clinical relevance.

## Background

Spontaneous aneurysmal subarachnoid hemorrhage (SAH), very often from the rupture of an intracranial aneurysm, is a neurological emergency associated with high morbidity and mortality worldwide [1–3]. The sudden intracranial bleeding causes a dramatic increase of intracranial pressure (ICP), a drop of cerebral perfusion pressure (CPP), and a cerebral blood flow (CBF) reduction. This transient global cerebral ischemia may have serious consequences [2–5]. After SAH, the main goal of neurocritical care is to prevent secondary brain injury [4, 6]. In particular, aneurysmal rebleeding, occurring more frequently within the first 24 h after SAH, increases the risk of mortality and poor clinical outcome [2, 3, 7, 8]. Rapid aneurysm securing by endovascular or neurosurgical treatment is therefore essential [9, 10]. Definitive aneurysm treatment and ventricular drain, which is often necessary, can only be performed in a specialized center. As a consequence, patients admitted to nonspecialized centers need to be transferred to a referral hospital as soon as possible [9, 10].

At present, only few aspects of SAH management are supported by high-quality studies, and most management principles are based on weak evidence [6, 9, 10]. This is especially true regarding the management of SAH patients in the hospital without neurosurgical/neurointerventional facilities and the transfer to referral hospitals. To address this issue, we created a consensus of experts endorsed by the Italian Society of Anesthesia and Intensive Care (SIAARTI). The specific aim of this consensus was to provide recommendations on the following:

- The early management of SAH patients admitted to the hospital without neurosurgical/neurointerventional facilities and
- The transfer to referral hospital for aneurysm’s definitive treatment.

## Methods

### Panel selection and governance

The multidisciplinary consensus panel was composed of 16 voting physicians, a methodologist (CR) and two advisory board members (GC and NS) selected for their established clinical and scientific expertise in the management of SAH patients with different specializations: anesthesia/intensive care, neurosurgery, and interventional neuroradiology. The SIAARTI endorsed the project and supervised the methodology and structure of the consensus. The consensus was led by a steering committee (EP, FR and CR) who (a) conceived the project establishing the objectives, (b) organized and set the agenda for the voting of recommendations, (c) ensured communications within the panel, and (d) drafted the report.

### Delphi process

Following a non-systematic review of the literature, the steering committee identified the domains and generated a list of questions to be addressed by the panel. The initial list of statements was formulated and distributed to the panelists 1 week prior to every Delphi round in order to allow modifications or additional statements. The modified iterative Delphi process was conducted using online tools [11, 12]. In a preliminary step, questions were circulated. Based on the initial answers and on comments/suggestions by the voting members, ambiguities and inconsistencies in the questionnaire were identified and corrected, generating a refined question set for subsequent voting rounds. We used an iterative approach; members were informed of the degree of consensus reached on the initial question round and asked to reconsider agreement or disagreement. Then, based on the answers collected at the third stage, statements for practical advice were proposed. The objective was to reach consensus, not necessarily unanimity.

The analysis of voting results was performed by a nonvoting experienced methodologist (CR). A decision rule was predefined to ascertain the degree of consensus required to provide a recommendation. Statements were classified as a *strong recommendation*, *weak recommendation*, and *no recommendation* when respectively > 85%, 75–85%, and <75% of votes were in favor.

### Terminology

In the following statements, a center without appropriate resources for aneurysm treatment both in personnel (cerebrovascular neurosurgeons, endovascular neuroradiologists, and neurointensivists) and/or technological facilities for diagnosis and therapy is indicated as Spoke. A specialized, high-volume center with all necessary facilities and staff available 24 h/day is, on the contrary, considered as Hub.

These terms refer to a well-coordinated “Hub and Spoke” system, where communications and cooperation are optimal. Our recommendations, however, may apply also to different situations, where a system has not yet been implemented. In that case, Hub and Spoke are simply indicating hospitals with different equipment and staff.

## Results

**Fig. 1:**
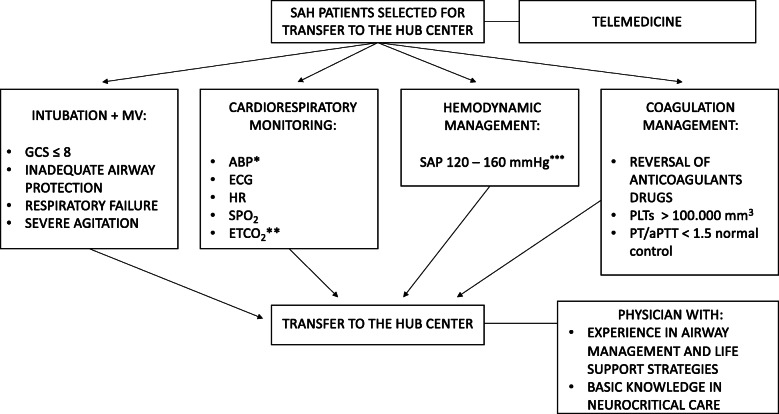
Consensus flow chart. ^*^Invasive in poor-grade SAH patients. ^**^In case of invasive mechanical ventilation. ^***^According to patient’s clinical history (i.e., arterial hypertension) and/or radiological signs of intracranial hypertension; Abbreviations: *MV* mechanical ventilation, *GCS* Glasgow Coma Scale, *ABP* arterial blood pressure, *ECG* electrocardiogram, *HR* heart rate, *SpO*_*2*_ peripheral oxygen saturation, *ETCO*_*2*_ end-tidal carbon dioxide, *SAP* systolic arterial pressure, *PLTs* platelets, *PT* prothrombin time, *aPTT* activated partial thromboplastin time

The consensus provided a total of 14 statements. Consensus was reached on 13 topics, leading to 13 corresponding recommendations (Table 1): 11 were *strong recommendations,* endorsed by more than 85% of participants, while 2 were *weak recommendations*, supported by 75–85%. The consensus flow chart was reported in Fig. 1. We were unable to reach consensus in one case. The consensus recommendations are listed below with the percentage of agreement.

**Table 1:** List of consensus recommendations

No.	Recommendation	Level
1	We recommend that all salvageable spontaneous SAH patients (i.e., patients who may recover, at least to some extent, with appropriate treatment) admitted in a Spoke center be rapidly transferred to a Hub center after hemodynamic and respiratory stabilization.	***Strong recommendation***
2	We recommend using a telemedicine service for image transfer from the Spoke to the Hub center.	***Strong recommendation***
3	We recommend that the transfer of SAH patients should be performed by a physician with: experience in advanced airway management and life support strategies and basic knowledge in neurocritical care (i.e., medical management of cerebral swelling, herniation).	***Strong recommendation***
4	We recommend sedation, intubation and mechanical ventilation for SAH patients in coma (Glasgow Coma Scale (GCS) score ≤ 8) and/or with inadequate airway protection or respiratory failure.	***Strong recommendation***
5	We recommend sedation, intubation, and mechanical ventilation also for SAH cases with severe agitation, if this persists despite mild sedation and pain control.	***Weak recommendation***
6	We recommend, in poor-grade SAH patients needing transfer to the Hub center, an invasive monitoring of arterial blood pressure (ABP) in addition to the standard cardiorespiratory monitoring (electrocardiogram (ECG), heart rate (HR), peripheral oxygen saturation (SpO_2_) and end-tidal carbon dioxide (ETCO_2_)).	***Strong recommendation***
7	We recommend, to avoid aneurysmal rebleeding and to ensure an adequate CPP, the maintenance of systolic arterial pressure (SAP) between 120 and 160 mmHg. It is also reasonable to individualize the target considering patient’s clinical history (i.e., arterial hypertension) and/or radiological signs of intracranial hypertension.	***Strong recommendation***
8	We recommend the maintenance of SAP values close to the lower limit (120 mmHg) in SAH patients without a history of arterial hypertension and/or radiological signs of elevated ICP.	***Strong recommendation***
9	We recommend the maintenance of SAP values close to the upper limit (160 mmHg), avoiding fluctuations, in SAH patients with a history of arterial hypertension and/or radiological signs of elevated ICP.	***Strong recommendation***
10	We recommend the maintenance of a platelet (PLT) count > 100,000/mm^3^ in all salvageable SAH patients, possibly candidates for neurosurgical intervention.	***Strong recommendation***
11	We recommend maintaining a prothrombin time (PT)/ activated partial thromboplastin time (aPTT) value < 1.5 the normal control in all salvageable SAH patients.	***Strong recommendation***
12	We recommend the early reversal of anticoagulants drugs in all salvageable SAH patients.	***Strong recommendation***
13	We recommend against the utilization of routine tranexamic acid for a short-term therapy before aneurysm treatment to prevent rebleeding of cerebral aneurysm/s.	***Weak recommendation***
14	We are unable to provide any recommendation regarding the use of routine seizure prophylaxis in all SAH patients.	***No recommendation***

Abbreviations: *SAH* subarachnoid hemorrhage, *ICP* intracranial pressure, *GCS* Glasgow Coma Scale, *ABP* arterial blood pressure, *ECG* electrocardiogram, *HR* heart rate, *SpO*_*2*_ peripheral oxygen saturation, *ETCO*_*2*_ end-tidal carbon dioxide, *CPP* cerebral perfusion pressure, *SAP* systolic arterial pressure, *PLTs* platelets, *PT* prothrombin time, *aPTT* activated partial thromboplastin time.

### Recommendation 1

We recommend that all salvageable spontaneous SAH patients (i.e., patients who may recover, at least to some extent, with appropriate treatment) admitted in a Spoke center be rapidly transferred to a Hub center after hemodynamic and respiratory stabilization (agreement: 87%, strong recommendation).

### Recommendation 2

We recommend using a telemedicine service for image transfer from the Spoke to the Hub center (agreement: 100%, strong recommendation).

### Recommendation 3

We recommend that the transfer of SAH patients should be performed by a physician with:

- Experience in advanced airway management and life support strategies and
- Basic knowledge in neurocritical care (i.e., medical management of cerebral swelling, herniation) (agreement: 93%, strong recommendation)

### Recommendation 4

We recommend sedation, intubation, and mechanical ventilation for SAH patients in coma (Glasgow Coma Scale (GCS) score ≤ 8) and/or with inadequate airway protection or respiratory failure (agreement: 93%, strong recommendation)

### Recommendations 5

We recommend sedation, intubation, and mechanical ventilation also for SAH cases with severe agitation, if this persists despite mild sedation and pain control (agreement: 81.5%, weak recommendation).

### Recommendation 6

We recommend, in poor-grade SAH patients needing transfer to the Hub center, an invasive monitoring of arterial blood pressure (ABP) in addition to the standard cardiorespiratory monitoring (electrocardiogram (ECG), heart rate (HR), peripheral oxygen saturation (SpO_2_), and end-tidal carbon dioxide (ETCO_2_)) (agreement: 93%, strong recommendation).

### Recommendation 7

We recommend, to avoid aneurysmal rebleeding and to ensure an adequate CPP, the maintenance of systolic arterial pressure (SAP) between 120 and 160 mmHg. It is also reasonable to individualize the target considering patient’s clinical history (i.e., arterial hypertension) and/or radiological signs of intracranial hypertension (agreement: 93%, strong recommendation).

### Recommendation 8

We recommend the maintenance of SAP values close to the lower limit (120 mmHg) in SAH patients without a history of arterial hypertension and/or radiological signs of elevated ICP (agreement: 87%, strong recommendation).

### Recommendation 9

We recommend the maintenance of SAP values close to the upper limit (160 mmHg), avoiding fluctuations, in SAH patients with a history of arterial hypertension and/or radiological signs of elevated ICP (agreement: 87%, strong recommendation).

### Recommendation 10

We recommend the maintenance of a platelet (PLT) count > 100,000/mm^3^ in all salvageable SAH patients possibly candidates for neurosurgical intervention (agreement: 93%, strong recommendation).

### Recommendation 11

We recommend maintaining a prothrombin time (PT)/ activated partial thromboplastin time (aPTT) value < 1.5 the normal control in all salvageable SAH patients (agreement: 93%, strong recommendation).

### Recommendation 12

We recommend the early reversal of anticoagulant drugs in all salvageable SAH patients (agreement: 93%, strong recommendation).

### Recommendation 13

We recommend against the utilization of routine tranexamic acid for a short-term therapy before aneurysm treatment to prevent rebleeding of cerebral aneurysm/s (agreement: 81%, weak recommendation)

### Recommendation 14

We are unable to provide any recommendation regarding the use of routine seizure prophylaxis in all SAH patients (agreement: 69%, no recommendation).

## Discussion

### Patient transfer to the Hub center

Aneurysmal rebleeding is more frequently observed during the first 24 h after SAH and is associated with high mortality rates [2, 3, 7, 8]. Therefore, after SAH diagnosis, it is of paramount importance to prevent aneurysm rebleeding by transferring the patient to a specialized center to allow the rapid treatment of the aneurysm [9, 10]. The management of SAH patients in high-volume centers, with experienced staff (neurovascular surgeons, endovascular neuroradiologists and neurocritical care specialists), is associated with improved neurological outcomes [9, 10, 13, 14]. As recommended also by recent guidelines [15], the transfer should be performed after cardiorespiratory stabilization. Some panel members, during the consensus development, suggested that a quick, clear, and complete communication (regarding medical information, transport timing, etc.) between hospitals and within the Hub center (activation of neurosurgeons, neuroradiologists, and neurointensivists) is advisable in these cases. The availability of shared protocols could be helpful in this regard and should be encouraged.

The transfer of radiological images by a web-based software allows neurosurgical consultation between hospitals. This procedure, also preventing unnecessary transfers, is considered life and time-saving as well as cost-effective [16, 17]. All panel members agreed on this point. Therefore, telemedicine should be encouraged in this setting.

SAH patients can be admitted to the Spoke hospital with a range of neurological severity (from headache to coma) and with a number of extracranial problems (i.e., neurogenic pulmonary edema, Takotsubo cardiomyopathy) [2, 3, 6]. SAH patients can also deteriorate at any time during the transfer (rebleeding, seizures, etc.). Therefore, as suggested also by other guidelines [15], these patients should be accompanied during the transfer by a physician with expertise in airway management, life support strategies, and basic knowledge about neurocritical care. Considering the abovementioned points and also according to recent guidelines [15], an adequate cardiorespiratory monitoring seems to be fundamental for the safety of the patients. In particular, invasive BP monitoring, allowing a precise and continuous BP estimation, is preferable, especially in unstable or poor-grade SAH. In addition, during the consensus rounds, some panelists suggested that a standard and basic cardiorespiratory monitoring (ECG, HR, SpO_2_, and noninvasive BP (NIBP)) should be utilized during the transfer also in mild cases. However, the placement of an arterial line should not delay excessively patient’s transfer, and NIBP can be considered a valid alternative in case of difficult arterial puncture.

### Airway management

Comatose SAH patients (GCS ≤ 8), unable to protect their airway and/or cardiorespiratory compromise, require tracheal intubation and mechanical ventilation [6, 16]. These maneuvers should also be reserved to patients who remain very agitated despite mild sedation and pain control [6, 16]. Tracheal intubation needs to be performed carefully, with adequate analgo-sedation, to avoid increase in ABP that can facilitate rebleeding or severe hypotension with possible cerebral hypoperfusion [16]. The panelists, in case of mild sedation and pain control, suggested the utilization of drugs with a short half-life and are easily titratable in order to allow a reliable neurological examination.

### Hemodynamic and coagulation management for rebleeding prevention

Important ABP fluctuations can be dangerous after SAH. High values of ABP can increase the risk of rebleeding (by increasing transmural pressure), and low values can exacerbate secondary brain injury reducing CPP especially in case of intracranial hypertension [6, 18, 19]. Moreover, it is important to keep in mind that in case of chronic arterial hypertension, the cerebral autoregulation curve is shifted to the right [20], and these patients are more likely to experience cerebral hypoperfusion, especially in case of elevated ICP [10, 20]. While there are various indications with regard to maintaining an SAP < 160 mmHg, little is known about the minimum value of SAP to be tolerated [9]. The panel agreed, as a safe lower SAP, a value of 120 mmHg being the upper limit of normal SAP [20]. ABP values, before aneurysm treatment, should be also individualized considering patient’s past medical history and the risk of intracranial hypertension.

Coagulopathy can promote rebleeding [21]. Moreover, some SAH patients may be in therapy with antithrombotic and anticoagulant drugs for preexisting pathologies [18, 21]. In this case, a PLT count greater than 100,000/mm^3^ (especially in patients needing neurosurgical intervention) as well as a PT/aPTT value < 1.5 the normal control seems to be reasonable to prevent complications. These recommendations, in addition to the reversal of anticoagulant drugs, are in accordance with previous recommendations [21, 22]. In addition, any physician involved in the management of these patients should be aware of the strategies regarding the reversal of anticoagulation, also considering the increase in the utilization of novel oral anticoagulants (NOACs) [18, 21]. The utilization of point-of-care (POC) tests (i.e., thromboelastometry (TEG) and rotational thromboelastography (ROTEM)), although not easily available (especially in peripheral hospital), can be useful to guide the reversal of these drugs [23]. The most recent American Heart Association (AHA) guidelines for SAH management consider reasonable a short-term (< 72 h) therapy with tranexamic acid (1 g every 6 h) or aminocaproic acid to reduce the risk of early rebleeding especially in patients with an unavoidable delay in aneurysm obliteration and in the absence of medical contraindications considering the increased risk of thrombosis [9]. Recently, the data from the ultra-early tranexamic acid after subarachnoid hemorrhage (ULTRA) study (a multicenter randomized controlled trial) showed that an ultra-early (as soon as possible), short-term tranexamic acid treatment (1 g bolus followed by a continuous infusion of 1 g terminated immediately before aneurysm treatment, or 24 h after start of the medication) did not improve clinical outcome at 6 months [24]. In this study, the median time from computed tomography (CT) scan to the start of aneurysm treatment was 14 h. Our recommendation refers mainly to this study. However, some panel experts suggested that, unfortunately, in some centers, the aneurysm treatment cannot be performed within 24 h or as fast as in the ULTRA study.

Finally, we found no consensus on the prophylactic use of antiepileptic therapy in patients with SAH. While the most recent guidelines suggest to avoid the prophylactic administration of antiepileptic drugs in SAH patients [9], part of the consensus panel consider it as clinical practice, especially in poor-grade SAH and in case of clinical or radiological features which increase the risk of seizures (i.e., aneurysm’s location, presence of hematoma).

### Limitations

These recommendations are based on the clinical expertise and knowledge of the participants. We deliberately did not base our statements on systematic literature reviews because of the lack of evidence (previously underlined) and in favor of simple, basic topics that are rarely subject to investigation.

Important aspects of diagnosis and care are not covered: for the sake of simplicity, we did not address important topics as the neurologic examination, the diagnosis of SAH, diagnosis and treatment of pulmonary and cardiac consequences of SAH, etc.. Accordingly, we did not explore the design of a well-organized Hub and Spoke system.

## Conclusions

The aim of this consensus was to create recommendations to support clinician’s decision-making in the management of SAH patients in Spoke hospitals. Due to insufficient evidence, our recommendations do not represent a mandatory standard of practice but are suggestions by clinicians to clinicians.

## Abbreviations

SAH: Subarachnoid hemorrhage
SIAARTI: Italian Society of Anesthesia and Intensive Care
ICP: Intracranial pressure
CBF: Cerebral blood flow
DCI: Delayed cerebral ischemia
GCS: Glasgow Coma Scale
ABP: Arterial blood pressure
BP: Blood pressure
ECG: Electrocardiogram
HR: Heart rate
SpO_2_: Peripheral oxygen saturation
ETCO_2_: End-tidal carbon dioxide
CPP: Cerebral perfusion pressure
SAP: Systolic arterial pressure
PLTs: Platelets
PT: Prothrombin time
aPTT: Activated partial thromboplastin time
NIPB: Noninvasive ABP
POC: Point-of-care
TEG: Thromboelastometry
ROTEM: Rotational thromboelastography
AHA: American Heart Association
ULTRA: Ultra-early tranexamic acid after SAH
CT: Computed tomography

## References

[1] Etminan N, Chang HS, Hackenberg K, de Rooij NK, Vergouwen MDI, Rinkel GJE, Algra A (2019). Worldwide incidence of aneurysmal subarachnoid hemorrhage according to region, time period, blood pressure, and smoking prevalence in the population: a systematic review and meta-analysis. JAMA Neurol.

[2] Macdonald RL, Schweizer TA (2017). Spontaneous subarachnoid haemorrhage. Lancet.

[3] Lawton MT, Vates GE (2017). Subarachnoid hemorrhage. N Engl J Med.

[4] Amodio S, Bouzat P, Robba C, Taccone FS (2020). Rethinking brain injury after subarachnoid hemorrhage. Crit Care.

[5] Rass V, Helbok R (2019). Early brain injury after poor-grade subarachnoid hemorrhage. Curr Neurol Neurosci Rep.

[6] Muehlschlegel S (2018). Subarachnoid hemorrhage. Continuum (Minneap Minn).

[7] Stienen MN, Germans M, Burkhardt JK, Neidert MC, Fung C, Bervini D, Zumofen D, Röthlisberger M, Marbacher S, Maduri R, Robert T, Seule MA, Bijlenga P, Schaller K, Fandino J, Smoll NR, Maldaner N, Finkenstädt S, Esposito G, Schatlo B, Keller E, Bozinov O, Regli L, Swiss SOS Study Group (2018). Predictors of in-hospital death after aneurysmal subarachnoid hemorrhage: analysis of a nationwide database (Swiss SOS [Swiss study on aneurysmal subarachnoid hemorrhage]). Stroke.

[8] Germans MR, Coert BA, Vandertop WP, Verbaan D (2014). Time intervals from subarachnoid hemorrhage to rebleed. J Neurol.

[9] Connolly ES, Rabinstein AA, Carhuapoma JR, Derdeyn CP, Dion J, Higashida RT, Hoh BL, Kirkness CJ, Naidech AM, Ogilvy CS, Patel AB, Thompson BG, Vespa P, American Heart Association Stroke Council, Council on Cardiovascular Radiology and Intervention, Council on Cardiovascular Nursing, Council on Cardiovascular Surgery and Anesthesia, Council on Clinical Cardiology (2012). Guidelines for the management of aneurysmal subarachnoid hemorrhage: a guideline for healthcare professionals from the American Heart Association/American Stroke Association. Stroke.

[10] Steiner T, Juvela S, Unterberg A, Jung C, Forsting M, Rinkel G, European Stroke Organization (2013). European Stroke Organization guidelines for the management of intracranial aneurysms and subarachnoid haemorrhage. Cerebrovasc Dis.

[11] Hopkins PM, Cooke PJ, Clarke RC, Guttormsen AB, Platt PR, Dewachter P, Ebo DG, Garcez T, Garvey LH, Hepner DL, Khan DA, Kolawole H, Kopac P, Krøigaard M, Laguna JJ, Marshall SD, Mertes PM, Rose MA, Sabato V, Savic LC, Savic S, Takazawa T, Volcheck GW, Voltolini S, Sadleir PHM (2019). Consensus clinical scoring for suspected perioperative immediate hypersensitivity reactions. Br J Anaesth.

[12] Fitch K, Bernstein SJ, Aguilar MD, Burnand B, LaCalle JR, Lazaro P, van heet Loo M, McDonnell J, Vader JP, Kahan JP (2001). The RAND/UCLA appropriateness method user’s manual.

[13] McNeill L, English SW, Borg N, Matta BF, Menon DK (2013). Effects of institutional caseload of subarachnoid hemorrhage on mortality: a secondary analysis of administrative data. Stroke.

[14] Josephson SA, Douglas VC, Lawton MT, English JD, Smith WS, Ko NU (2010). Improvement in intensive care unit outcomes in patients with subarachnoid hemorrhage after initiation of neurointensivist co-management. J Neurosurg.

[15] Nathanson MH, Andrzejowski J, Dinsmore J, Eynon CA, Ferguson K, Hooper T, Kashyap A, Kendall J, McCormack V, Shinde S, Smith A, Thomas E (2020). Guidelines for safe transfer of the brain-injured patient: trauma and stroke, 2019: guidelines from the Association of Anaesthetists and the Neuro Anaesthesia and Critical Care Society. Anaesthesia.

[16] Tatlisumak T, Soinila S, Kaste M (2009). Telestroke networking offers multiple benefits beyond thrombolysis. Cerebrovasc Dis.

[17] Kreutzer J, Akutsu H, Fahlbusch R, Buchfelder M, Nimsky C (2008). Teleradiology in neurosurgery: experience in 1024 cases. J Telemed Telecare.

[18] Sharma D (2020). Perioperative management of aneurysmal subarachnoid hemorrhage. Anesthesiology.

[19] Tang C, Zhang TS, Zhou LF (2014). Risk factors for rebleeding of aneurysmal subarachnoid hemorrhage: a meta-analysis. PLoS One.

[20] Taler SJ (2018). Initial treatment of hypertension. N Engl J Med.

[21] Frontera JA, Lewin JJ, Rabinstein AA, Aisiku IP, Alexandrov AW, Cook AM, Del Zoppo GJ, Kumar M, Peerschke EI, Stiefel MF, Teitelbaum JS, Wartenberg KE, Zerfoss CL (2016). Guideline for reversal of antithrombotics in intracranial hemorrhage: executive summary. A statement for healthcare professionals from the Neurocritical Care Society and the Society of Critical Care Medicine. Crit Care Med.

[22] Hess AS, Ramamoorthy J, Hess JR (2021). Perioperative platelet transfusions. Anesthesiology.

[23] Kvint S, Schuster J, Kumar MA (2017). Neurosurgical applications of viscoelastic hemostatic assays. Neurosurg Focus.

[24] Post R, Germans MR, Tjerkstra MA, Vergouwen MDI, Jellema K, Koot RW, Kruyt ND, Willems PWA, Wolfs JFC, de Beer FC, Kieft H, Nanda D, van der Pol B, Roks G, de Beer F, Halkes PHA, Reichman LJA, Brouwers PJAM, van den Berg-Vos RM, Kwa VIH, van der Ree TC, Bronner I, van de Vlekkert J, Bienfait HP, Boogaarts HD, Klijn CJM, van den Berg R, Coert BA, Horn J, Majoie CBLM, Rinkel GJE, Roos YBWEM, Vandertop WP, Verbaan D, ULTRA Investigators (2021). Ultra-early tranexamic acid after subarachnoid haemorrhage (ULTRA): a randomised controlled trial. Lancet.

